# Rigorous genetic diagnosis review in natural history studies

**DOI:** 10.1186/s13023-026-04317-2

**Published:** 2026-04-28

**Authors:** Amy Pizzino, Kaley Arnold, Emma Wiener, Kayla Muirhead, Johanna Schmidt, Carlos A. Dominguez-Gonzalez, Pooja Banglorewala, Jamie L. Fraser, Maura Ruzhnikov, Julie S. Cohen, Omar Sherbini, Rachel Logan, Francesco Gavazzi, Anjana Sevagamoorthy, Ariel Vincent, Russell D’Aiello, Adeline Vanderver

**Affiliations:** 1 Division of Neurology, Children’s Hospital of Philadelphia, Philadelphia, PA USA; 2 Department of Pediatrics, Perelman School of Medicine, University of Pennsylvania, Philadelphia, PA USA; 3 Ambry Genetics, Aliso Viejo, CA USA; 4 Division of Genetics and Metabolism, Department of Pediatrics, Rare Disease Institute, Children’s National Hospital, Washington, DC USA; 5 Department of Neurology and Developmental Medicine, Kennedy Krieger Institute, Baltimore, MD USA; 6 Departments of Neurology and Genetic Medicine, Johns Hopkins University School of Medicine, Baltimore, MD USA; 7 Division of Neurosciences, Children’s Healthcare of Atlanta, Atlanta, GA USA; 8 Department of Neurology, Perelman School of Medicine, University of Pennsylvania, Philadelphia, PA USA; 9 Clinical Research Support Office, Children’s Hospital of Philadelphia, Philadelphia, PA USA; 10 Department of Biomedical & Health Informatics, Children’s Hospital of Philadelphia, Philadelphia, PA USA; 11 3615 Civic Center Blvd, Philadelphia, PA 19104 USA

**Keywords:** Leukodystrophy, Biorepository, Natural history, Diagnosis confirmation, Genetic testing

## Abstract

**Background:**

Leukodystrophies are a clinically and genetically heterogeneous group of diseases characterized by white matter abnormalities on brain magnetic resonance imaging. Clinical, biochemical, molecular, and/or neuroimaging findings collectively support the diagnosis confirmation. The heterogeneous and overlapping clinical presentations of different leukodystrophies and non-diagnostic molecular testing pose a significant challenge to establishing a definitive diagnosis in these rare diseases. The Myelin Disorders Biorepository Project is an observational research program that aims to establish new tests to diagnose leukodystrophies and describe the natural history of these disorders. Ensuring an accurate diagnosis is critical to the goals of this project, and this paper aims to describe the rigorous diagnostic review and confirmation process which was developed.

**Results:**

We present a diagnosis review process that contributes to an accurate diagnosis for participants enrolled in this study. Board-certified genetic counselors with expertise in these disorders audit medical records to carefully assess each enrolled participant’s clinical, biochemical, and molecular features. A scale of diagnostic categories is assigned based on the record review, and a team of leukodystrophy physician experts consults for cases that require further characterization or clarification.

**Conclusions:**

This robust review process has resulted in a database of individuals with verified diagnoses that may be easily queried for inclusion in appropriate natural history studies and/or treatment trials. This is a model framework that may be adapted and implemented by other rare disease groups.

## Background

Leukodystrophies are a clinically and genetically heterogeneous group of rare diseases characterized by central nervous system (CNS) white matter/myelin abnormalities [1]. Although individual leukodystrophies range from rare to ultra-rare, cumulatively, leukodystrophies affect 1 in 4,700 live births [2]. Given the severity, morbidity, and mortality of these disorders, a critical need exists to improve diagnostic accuracy and ultimately develop effective therapies. As with other rare disorders, research on these conditions requires cohorts of patients larger than a single institution or practitioner can provide to increase the statistical power and translational potential of studies [3].

The Global Leukodystrophy Initiative Clinical Trials Network (GLIA-CTN) is a research consortium dedicated to advancing the diagnosis, treatment, and management of leukodystrophies [4]. It utilizes a shared research infrastructure to collect and analyze robust clinical data and biological specimens. The Myelin Disorders Biorepository Project (MDBP) is the core database developed over 20 years ago to collect clinical and molecular data for affected participants followed across a network of nearly two dozen domestic clinical research sites participating in the GLIA-CTN.

Biorepositories such as these are essential tools for rare disease research and provide organization of patient information to facilitate significant research and improve clinical care [5]. The European Union Committee of Experts on Rare Diseases (EUCERD) published guidelines for rare disease repositories in 2013, emphasizing the importance of database governance, interoperability, adaptability, and sustainability. However, no guidance on data quality or maintenance standards was provided [3]. Frequent re-evaluation of the data structure, alongside rigorous methods to maintain high quality and accuracy of the data, is essential [3, 6]. Additional difficulties in diagnosis lie in the lack of agreement in rare disease definitions and how to address variants of uncertain clinical significance [7].

Individuals with confirmed or suspected leukodystrophies are eligible to participate in the MDBP. Enrollment occurs either remotely or in the context of an in-person visit to a GLIA-CTN site. Upon enrollment, each participant is assigned a unique study identifier and dedicated chart within a secure REDCap database hosted centrally at the Children’s Hospital of Philadelphia, which serves as the Data Coordinating Center (DCC) for the GLIA-CTN. Based on complete clinical information, a diagnosis of an individual’s specific leukodystrophy is assigned. Given that data in MDBP is used for multiple research projects, a diagnosis confirmation is critically important. Different sub-cohorts for disease-specific research, natural history studies, or clinical trials may be extracted.

Leukodystrophies have significant phenotype variability, as well as overlapping clinical presentations, radiological features, and biochemical features; thus reaching a confirmed diagnosis remains inherently challenging [8, 9]. Clinical presentation, followed by MRI brain imaging to enable confirmation that white matter abnormalities are causing the clinical symptoms serve as the starting point for diagnosis [1]. Neuroimaging helps narrow the differential diagnosis by characterizing white matter abnormalities [10]. In cases where the MRI pattern and clinical features are strongly suggestive of a specific leukodystrophy, single gene or gene panel testing may be appropriate to achieve diagnosis. In all other cases, whole genome sequencing is recommended as a first-line genetic diagnostic test, accompanied by appropriate biochemical studies [1, 11]. When there is concordance between clinical presentation, molecular, neuroimaging, and biochemical testing, one can assign the diagnosis with a high degree of certainty [10, 12].

Due to the difficulties in diagnosis accuracy in rare disease, the aim of this paper is to outline a rigorous diagnostic review and confirmation process which was developed to ensure available records in an existing database (the MDBP) support accurate diagnoses.

## Methods

### Materials and methods

The Myelin Disorders Biorepository Project (MDBP) at the Children’s Hospital of Philadelphia (CHOP IRB #14-011236) is a multi-center protocol that supports all observational research conducted across the Global Leukodystrophy Initiative Clinical Trials Network (GLIA-CTN), which currently comprises a total of 23 domestic clinical research institutions. The REDCap databases supporting the protocol are hosted centrally at the Children’s Hospital of Philadelphia [13, 14]. REDCap (Research Electronic Data Capture) is a secure, web-based software platform designed to support data capture for research studies, providing (1) an intuitive interface for validated data capture; (2) audit trails for tracking data manipulation and export procedures; (3) automated export procedures for seamless data downloads to common statistical packages; and (4) procedures for data integration and interoperability with external sources.

The MDBP is a long-standing project that has been refined and standardized over the past two decades as new resources and technologies have become available. Participants are entered into the primary database as either an affected individual or a healthy control (family member), with branching logic to reflect the necessary data collection fields for affected individuals. A separate but linked database captures the molecular details of all participants; this is called the MDBP/GLIA-CTN Molecular Database (“MolDb”).

**Fig. 1:**
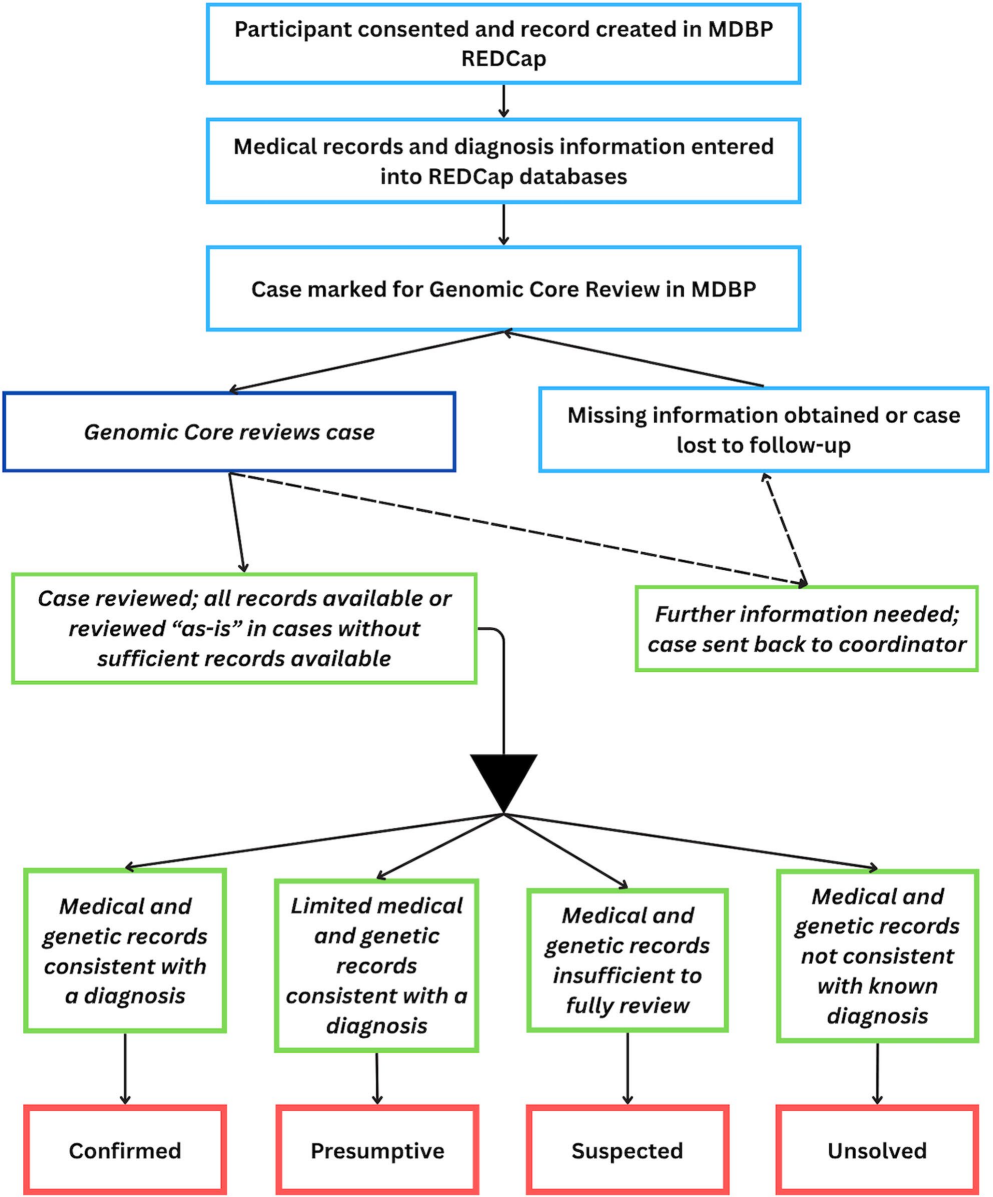
Diagnostic confirmation flow. Overview of the process by which participants are reviewed for appropriate diagnostic category. Boxes outlined in blue indicate roles performed by a clinical research coordinator and boxes outlined in green with italicized text are performed by members of the genomics core. Medical records encompass clinical notes, radiographical features, and biochemical testing, if applicable

Once a participant is coded as an affected individual, they are further divided into those with a diagnosis and those who do not yet have a known diagnosis (unsolved). As the repository grew, we recognized a need for a diagnostic confirmation process to improve accuracy. This formal process was initiated in 2021, and a standard operating procedure was created in 2023. The GLIA-CTN Genomics Core was formed during this time and includes a team of board-certified genetic counselors and physicians with expertise in leukodystrophies who developed this process and validated the functionality. Research coordinators also contributed to this process. An overview of this process is shown in Fig. 1.

We established criteria for case review and selection of the most suitable diagnostic category. This includes four primary criteria:

1. Clinical: Obtained through medical records or other appropriate clinical documentation review. The review evaluates the primary clinical features of disease, such as developmental regression or ataxia. Informal communication about the participant’s disease status, such as email, are not accepted as source documentation.
2. Molecular: Pathogenic or likely pathogenic variant(s) obtained from genetic testing reports or research reports. The initial pathogenicity assessment is based on the original lab report. Reclassification of a variant can be made manually via an amended or updated lab report, publication, or official genomic review (such as through a ClinGen Variant Curation Expert Panel). This review process currently does not include independent variant curation by the GLIA-CTN reviewers and relies on the laboratory report.
3. Radiographical: Review should ensure that MRI features support the diagnosis of the disease, as applicable. Clinical radiologic impressions were used, with a review conducted by a physician member of the GLIA-CTN as needed.
4. Biochemical: Disorders with biochemical phenotypes or diagnostic criteria. Some conditions may be diagnosed with a combination of radiographic features and biochemical testing without a molecular test, such as in metachromatic leukodystrophy. Whenever possible, efforts are made to obtain the biochemical laboratory report in addition to the molecular testing.

Once the diagnostic criteria was established, we developed categories based on the available source documentation. Four primary diagnostic categories exist, with subcategories in the presumptive category (Table 1).

**Table 1:** Diagnostic categories

Category		Criteria
Confirmed		Clinical and molecular source documentation exist and confirm the diagnosis. Molecular findings are classified as pathogenic or likely pathogenic in the genetic testing report.
Presumptive		Evidence exists to support the diagnosis but cannot fully confirm due to missing elements.
a.	Variant Reclassification Needed	Clinical and molecular source documentation exist and support the diagnosis but molecular report has one or more variants classified as a variant of uncertain clinical significance (VUS).
b.	Biochemical Results Only	Molecular testing has not been performed or the molecular report is not available for review; however, positive biochemical test results are available with clinical and radiologic features consistent with the diagnosis. A pseudodeficiency has been evaluated for when relevant to the extent possible. Alternative explanations for abnormal biochemical results have also been explored when relevant.
c.	Affected Family Member	Although limited medical records exist in the participant’s name, sufficient evidence exists to review and establish either a clinical or molecular diagnosis AND this individual has a similarly affected family member with a diagnosis that would qualify as Confirmed or Presumptive: Variant Reclassification Needed.
d.	Research-Based Testing Only	Diagnosis is supported by clinical presentation, and characteristic features on brain MRI but clinical molecular testing has not been done. Research-based genetic testing has identified a likely pathogenic or pathogenic variant(s).
e.	Incomplete Records	Although limited, medical records exist with enough evidence to review and establish either a clinical or molecular diagnosis.
f.	Incomplete Genotype	Single-allele variant (likely pathogenic, pathogenic, or VUS) identified in recessive disease gene when clinical phenotype is consistent with no second variant identified. Alternatively, biochemical testing is consistent with disease but genetic testing is negative.
g.	Genotype not consistent with described phenotype	Molecular testing revealed pathogenic or likely pathogenic variant(s) associated with a particular diagnosis; however, symptoms differ from current known/standard phenotypic presentation and may represent a new phenotypic presentation.
Suspected		There are insufficient records to review. This includes pre-enrollment cases who have provided consent but are in the process of finishing enrollment with records collection, cases that are lost to follow-up, as well as cases with records in a language other than English.
No Diagnosis (Unsolved)		Case does not have a known diagnosis, confirmed through review of available records by the above criteria.

There are four primary diagnostic categories with seven subcategories within the presumptive category to allow for detailed stratification

The review process begins with creating a study number assigned by a research coordinator who enrolls a participant. A research number is not assigned until all necessary consent documents have been obtained. Once consent is complete and records are received, or the case is deemed lost to follow-up, it is sent for review by the Genomics Core (Fig. 1). Automatic notifications via REDCap ensure that the case proceeds to the Genomics Core for review.

Once the Genomics Core reviewer receives a notification, they review the available source documents in the biorepository according to the criteria listed in table one. In addition to selecting the appropriate diagnostic category (Confirmed, Presumptive, Suspected, or Unsolved), the reviewer also indicates the time and date of the review and selects themselves from a dropdown menu as the most current reviewer. If the case has all available source documentation, the case is “Diagnosis Locked (no follow-up needed).” If additional source documentation is available and obtainable, the case is “Pending Follow-up (Information Needed).” The diagnostic category is selected for the best fit based on the available information at the time of review. This additional field allows the team to identify cases where extra information could be obtained. Re-reviewed cases are updated with the reviewer and the date/time of the most recent review. A final question captures unique cases that would not be eligible for natural history studies, such as cases with comorbid diagnoses.

**Fig. 2:**
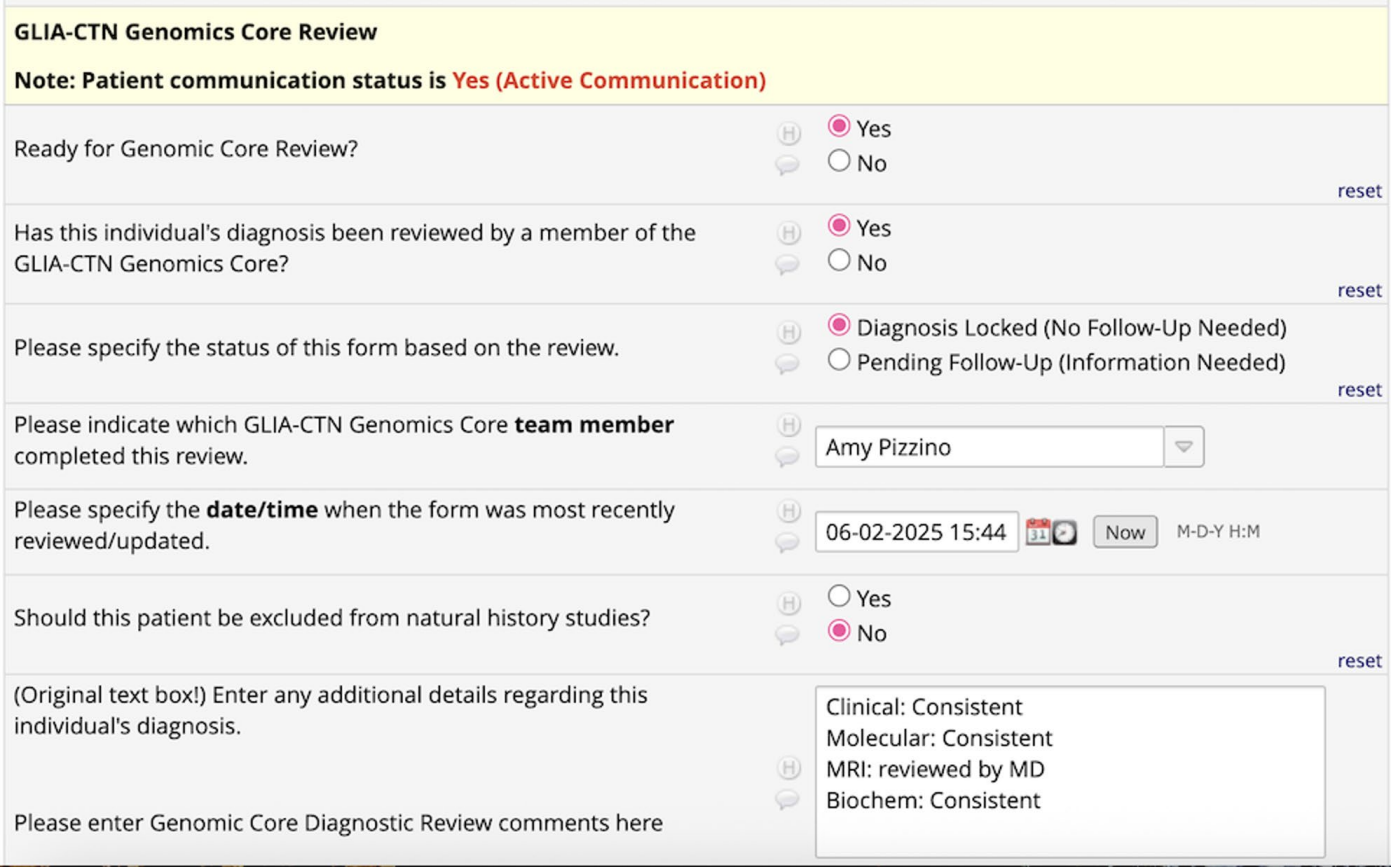
Screenshot of a reviewed confirmed case. This screenshot shows the REDCap page where the diagnosis review is recorded. The most current date and time of review as well as the Genomics Core member who performed the review are captured as well as the status of review. Notes specific to the case can be entered freely in the text box

Within the REDCap database, the information is captured in discrete questions to allow data sorting. A final text entry box allows for free text detailing the diagnosis not captured in preceding fields and includes a review summary (Fig. 2).

### Challenges in diagnostic review

The diagnostic review process may be complicated by the presence of dual diagnoses, differences in nomenclature used by reporting labs, and assessments of pathogenicity. This database captures up to three diagnoses related to leukodystrophy, defined as follows:

Primary Diagnosis: The confirmed, presumed, or suspected disease primarily responsible for the symptoms that qualified the individual for MDBP. Secondary/Tertiary Diagnosis: Any disease confirmed clinically and/or molecularly with symptoms that may overlap with, modify, or confound the primary diagnosis. Diagnoses lacking phenotypic overlap with the primary diagnosis (for example, hereditary cancer syndromes, etc.) are recorded in a separate database dedicated to the molecular data of MDBP participants.

To capture details on the variants identified, a molecular database was developed. This enables the reviewer to concentrate on the variants identified in each subject and filter for variants that are directly relevant to the primary, secondary, or tertiary diagnoses while noting additional variants that may have been identified in a subject. Variants related to the primary, secondary, or tertiary leukodystrophy are aligned with the Matched Annotation from the NCBI and EMBL-EBI (MANE) transcript based on the transcript reported in the laboratory report, if available. For cases with no report or no transcript listed, the variant is verified using the date of variant discovery, if known, NCBI/Ensembl, and a comparison of nucleotide and amino acid changes to identify the most likely transcript.

### Publications yielded using this rigorous diagnosis framework

To better understand the impact of this process, we identified publications that referenced the grant supporting MDBP, U54NS115052. We refined these to include only those published since the inception of the diagnosis review process in 2021. These papers were further reviewed and grouped by theme: natural history, outcome measures, treatment, novel gene-disease discovery, and others. Natural history was defined as papers describing the natural course of disease. They include genotype-phenotype correlation studies, papers that assessed specific symptoms or imaging features associated with a disease, and biomarker studies. Functional outcomes are defined as papers that use measures to evaluate a participant’s status and ability to perform activities of daily living. Treatment applies to papers that address novel or known treatments in leukodystrophy and includes pharmacological approaches and procedural treatments such as bone marrow transplants. Diagnosis references papers that describe a novel leukodystrophy-gene-disease relationship, as well as papers describing the diagnostic odyssey. Finally, papers that could not be grouped into one of the prior categories were categorized as others and generally capture review and consensus papers.

## Results

**Fig. 3:**
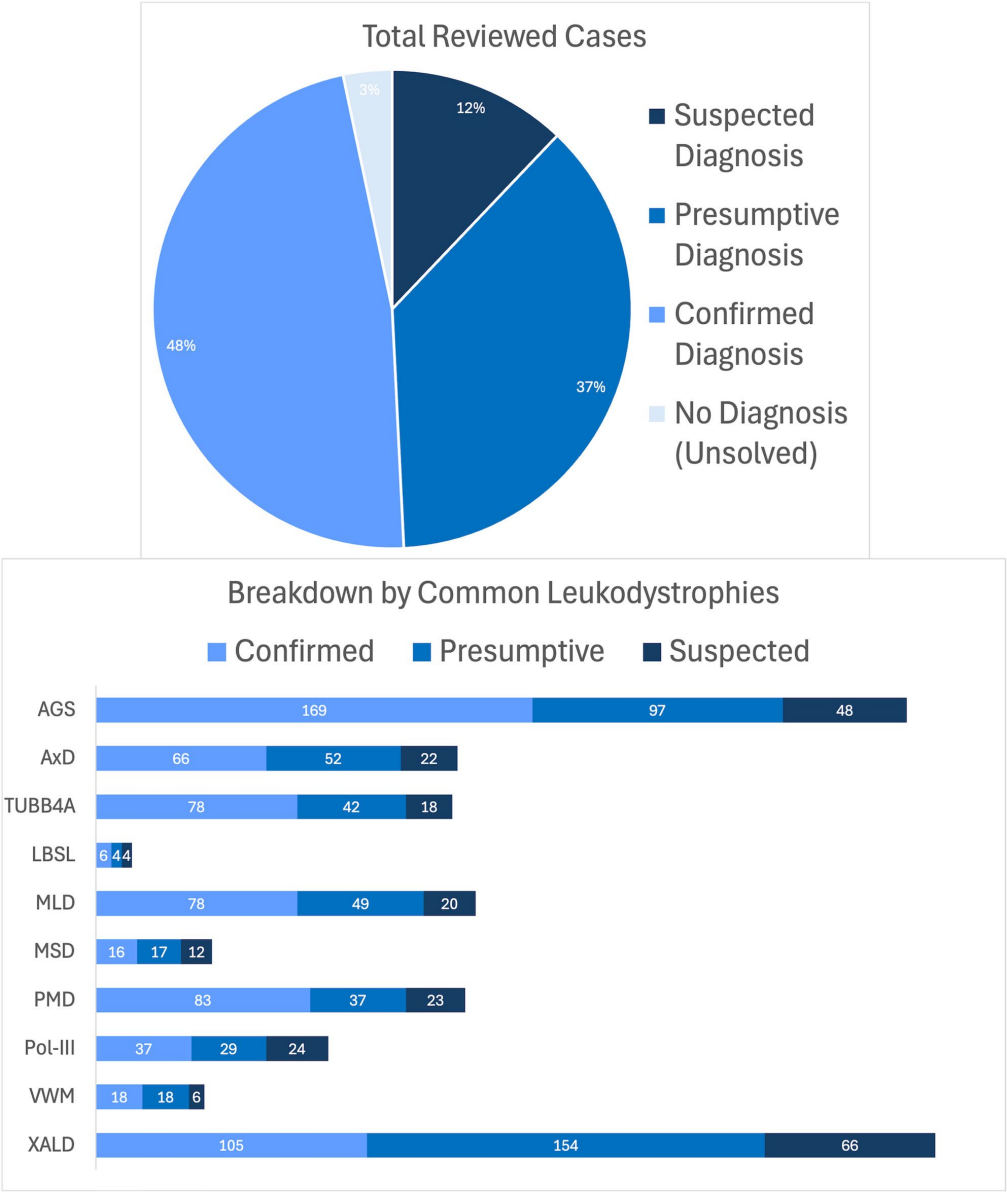
State of review by genomics core. a total of 2,000 cases have been reviewed by the genomics core using the diagnosis review process, with most cases reaching a confirmed or presumptive category. a detailed breakdown by the most common leukodystrophies is shown in the bar graph. note that this process was developed for the confirmation of diagnoses made, as such, the majority of enrolled cases without a diagnosis have not been reviewed

As of May 1, 2025, 3,524 cases have been enrolled in MDBP, and 2,000 of those cases have been reviewed using this process. These 2,000 participants reflect primarily diagnosed individuals within the biorepository, who were reviewed for confirmation of their diagnoses. Figure 3 outlines the total number of reviewed cases, segregated by diagnostic category and stratified by the most common leukodystrophies. Table 2 provides further detail related to diagnostic categories using examples involving Aicardi-Goutières Syndrome (AGS) and Metachromatic Leukodystrophy (MLD).

**Table 2:** State of review by genomics core with selected disease examples

All Diagnoses		Aicardi-Goutières Syndrome (AGS)		Metachromatic Leukodystrophy (MLD)	
Totals		Total AGS Cases		Total MLD Cases	
Cases Enrolled:	3524	Total AGS Cases	318	Total MLD Cases	152
Confirmed:	950	Confirmed:	169	Confirmed:	78
Suspected:	833	Suspected:	52	Suspected:	25
Presumptive:	749	Presumptive:	97	Presumptive:	49
Unsolved:	968	Unsolved:	0	Unsolved:	0
**Reviewed Totals**		**AGS Reviewed Totals**		**MLD Reviewed Totals**	
Cases reviewed by Genomics Core	2001	Cases reviewed by Genomics Core	314	Cases reviewed by Genomics Core	147
Confirmed	950	Confirmed	169	Confirmed	78
Suspected	242	Suspected	48	Suspected	20
Presumptive	743	Presumptive	97	Presumptive	49
Unsolved	66	Unsolved	0	Unsolved	0
**Reviewed Presumptive**		**AGS Reviewed Presumptive**		**MLD Reviewed Presumptive**	
Variant Reclassification Needed	274	Variant Reclassification Needed	47	Variant Reclassification Needed	11
Biochemical Results Only	55	Biochemical Results Only	0	Biochemical Results Only	10
Family Member Diagnosis	15	Family Member Diagnosis	3	Family Member Diagnosis	1
Research-Based Testing	43	Research-Based Testing	13	Research-Based Testing	0
Incomplete Records	314	Incomplete Records	16	Incomplete Records	26
Incomplete Genotype	24	Incomplete Genotype	18	Incomplete Genotype	1
Genotype not consistent with described phenotype	12	Genotype not consistent with described phenotype	0	Genotype not consistent with described phenotype	0
Total	737	Total	97	Total	49
Suspected, not yet reviewed:	591	Suspected, not yet reviewed:	4	Suspected, not yet reviewed:	5
		Eligible for Natural History:	232	Eligible for Natural History:	100

This table demonstrates stratification by confirmation status using examples of individual diseases, AGS and MLD. MLD has biochemical diagnostic testing, thus highlighting the value of subcategories within the presumptive diagnostic category. For example, participants eligible for a study requiring genetic confirmation can easily be identified with this process. Note that this process was developed for the confirmation of diagnoses made; as such, the majority of enrolled cases without a diagnosis have not been reviewed

The diagnostic review process facilitates the rapid and accurate identification of subjects eligible for inclusion in further studies, such as natural history studies. The GLIA-CTN currently supports seven natural history studies with current and ongoing data collection, three studies being built and preparing for data collection, and three studies still in development. Subjects are automatically added to these natural history databases based on their consent and diagnostic categories. Table 2 illustrates how the review process categorizes diagnoses and enables automatic inclusion in natural history databases.

**Fig. 4:**
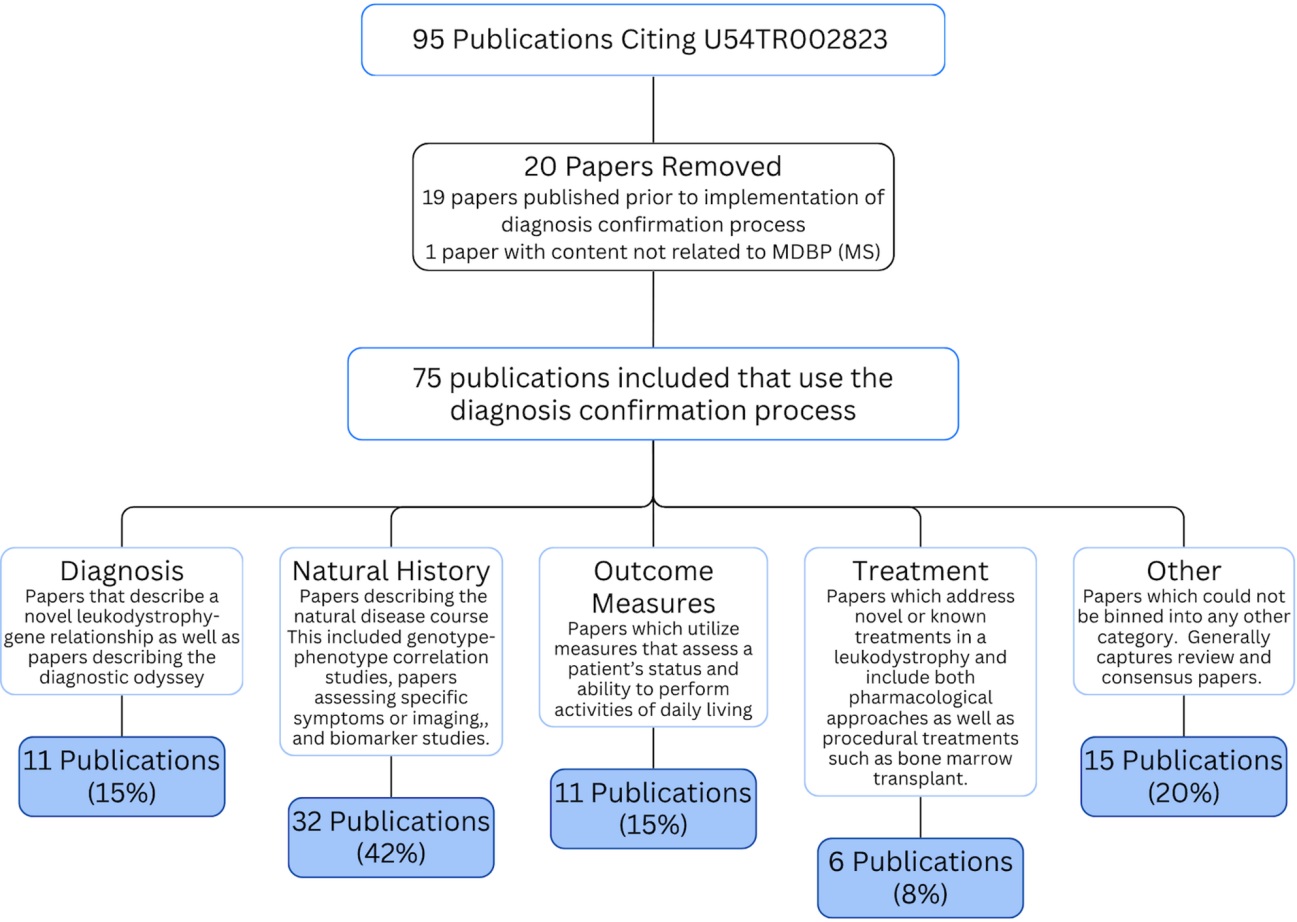
Publications depending on the diagnostic review process. publications which use data generated through MDBP as identified by credit to the GLIA-CTN U54NS115052 grant were filtered by year of publication as the diagnosis review process was formally implemented in 2021. we additionally reviewed to ensure publications used MDBP data. remaining publications were reviewed for common themes

A wide array of publications have benefited from this process, including studies about natural history, outcome measures, treatment, and novel disease-gene relationships. A total of 95 publications credit the U54NS115052 grant; 76 (80%) were published since the diagnosis confirmation process was implemented in 2021 (Fig. 4). One publication was removed due to a lack of MDBP content; the grant was mentioned only to acknowledge the lead author’s funding [15], leaving 75 (79%) total papers that utilized the diagnosis confirmation process [8, 15–89]. There were 11 (15%) publications related to diagnosis, including novel gene discoveries such as the linkage of *RNU4-2* to neurodevelopmental phenotypes [77] and the discovery of bi-allelic variants in *NOTCH3* leading to an early-onset leukoencephalopathy [53]. Thirty-two (42%) publications were related to the natural history of disease, both broadly, as in Adang et al. 2024, which described the curation of natural history data in rare diseases [72], as well as symptom-specific studies such as the craniofacial features associated with *POLR3* disorders [62]. Outcome measures, such as the validation of the GMFC-MLD in a *TUBB4A*-related disease [59], were described in 11 (15%) publications. Therapeutic advances in the leukodystrophies were described in 6 (8%) publications, including a phase 2–3 trial for adrenomyeloneuropathy [61]. Finally, 15(20%) publications were not categorized into prior categories and include consensus statements [27]. All of these publications rely on the accuracy of the reported diagnosis in the subjects referenced and highlight the impact of a diagnosis confirmation process with large databases.

## Discussion

Biorepositories play a crucial role in deepening our understanding of rare diseases and their natural history. The quality of research is directly linked to the quality of data. Here we describe a method designed to enhance the accuracy of clinically ascertained diagnoses documented in a large multi-center biorepository, ultimately improving the overall data quality. Furthermore, potential participants for future studies such as natural history or treatment trials may be efficiently stratified and identified for inclusion in subsequent studies. Although designed for the leukodystrophy population, this method may be adapted for other rare disease cohorts.

Natural history studies are fundamental in clinical research and development of clinical management guidelines and treatments (FDA [90]). The validity of natural history relies on an accurate diagnosis, which can be difficult in rare diseases where participant identification is challenging [72, 91, 92]. The process outlined here presents a method for identifying participants within a large rare-disease biorepository. This, in turn, has allowed for a more robust study of the natural history of more than seven rare leukodystrophies, as evidenced by the studies that currently exist within the MDBP.

Since the inception of this diagnostic confirmation process in 2021, the available treatment options and understanding of existing treatments for leukodystrophies have greatly expanded. New treatments include gene therapy for MLD [93, 94] and ALD [95]. On the horizon, the use of leriglitazone in the treatment of adrenomyeloneuropathy and adult-onset cerebral adrenoleukodystrophy has shown promise [61, 96]; treatment trial designs for Vanishing White Matter Disease (VWMD)have been published [55, 64]; and gene therapy in a mouse model of Multiple Sulfatase Deficiency (MSD) has shown potential [97]. Existing treatment for Cerebrotendinous Xanthomatosis (CTX) with the use of chenodiol has received FDA approval based on the results of the RESTORE trial [98]. Understanding of Janus kinase (JAK) inhibitors for the treatment of AGS has expanded to include additional ethnicities [99, 100] as well as subphenotypes [39]. A diagnostic confirmation process to readily identify eligible participants for ongoing studies facilitates rapid drug development and can make a significant difference in the lives of participants and their caregivers.

As with any process involving human subjects, complexities and challenges exist. In this process, we rely on the clinical judgment of trained genetics professionals, and subjectivity remains. Additionally, implementing any review process for an existing database presents challenges with data alignment and reviewing historical cases. Within MDBP, 741 cases have not yet been reviewed. While plans are in place to expedite the review of these cases, this largely depends on the work of the core reviewers.

The understanding of human genetic variation is growing, but uncertainty remains, even with guidelines from the American College of Medical Genetics (ACMG) and ClinGen [101, 102]. Within the MDBP database, variants are reviewed for accuracy, but pathogenicity relies predominantly on the original laboratory determination. The Genomics Core has been approved as a ClinVar submitter and actively submits relevant variants to ClinVar. Along with other experts, GLIA-CTN reviewers on our team are approved members of other large-scale biorepositories, serving on a ClinGen Gene Curation Expert Panel (GCEP) and Variant Curation Expert Panel (VCEP). The diagnostic confirmation process enables an accurate collection of participants with specific diseases and variants, which will help guide the GCEP and VCEP reviews.

## Conclusions

The implementation of a standardized diagnostic confirmation process that uses clinical, biochemical, molecular, and radiographical criteria improved data quality and enabled more efficient cohort identification for natural history studies, clinical trials, and other research efforts. Despite inherent diagnostic challenges due to the complexity of leukodystrophies, this framework has been essential for advancing research in the leukodystrophy space. As natural history studies and therapeutic opportunities for leukodystrophies continue to expand, such rigor in data curation is critical to supporting clinical and translational research in rare disease.

## Data Availability

All data generated or analyzed during this study are included in this published article. REDCap data dictionary available upon request.

## Abbreviations

ACMG: American College of Medical Genetics
FDA: Food and Drug Administration
GCEP: Gene Curation Expert Panel
GLIA-CTN: Global Leukodystrophy Initiative Clinical Trials Network
GMFC-MLD: Gross Motor Function Classification - Metachromatic Leukodystrophy
MDBP: Myelin Disorders Biorepository Project
VCEP: Variant Curation Expert Panel
